# In the Shadow of COVID-19: The Well-Being and Rights of People Living with Dementia in Finland During the Pandemic

**DOI:** 10.3233/JAD-221096

**Published:** 2023-04-18

**Authors:** Kaijus Ervasti, Toomas Kotkas, Mervi Issakainen, Minna Teiska, Anna Mäki-Petäjä-Leinonen

**Affiliations:** a Law School, University of Eastern Finland, Joensuu, Finland; b Faculty of Law, University of Helsinki, Helsinki, Finland; c Muistiliitto ry, Helsinki, Finland

**Keywords:** COVID-19, dementia, fundamental rights, law and society, pandemic, sociolegal studies, well-being

## Abstract

**Background:**

Exceptional circumstances such as the COVID-19 pandemic increase the risk for vulnerability among people living with dementia.

**Objective:**

This article discusses the well-being and rights of people living with dementia in Finland during the pandemic and analyses the legal framework covering the restrictions of their rights during that period.

**Methods:**

The empirical research comprises a survey of persons with dementia (*n* = 31) and their family members (*n* = 168). The participants completed a total of 13 survey items involving questions about their well-being during the pandemic, restrictions on freedom, access to services, information on pandemic regulations and guidelines as well as possible problems with authorities. The survey included both multiple choice and open-ended questions.

**Results:**

According to people with dementia and their family members, by spring 2021, the pandemic had reduced meaningful activities available to people living with dementia in Finland and decreased the number of meetings between them and other people. Many reported a decline in their physical and/or mental well-being or greater difficulty or delays in accessing social and health services. Over a third of respondents found that *the right to meet people* was restricted among people with dementia, and almost half of the respondents took the view that their *freedom of movement* was restricted. There were also major shortcomings in terms of information on restrictions.

**Conclusion:**

The results highlight the importance of bearing in mind the negative effects that restrictions on mobility, meeting other people and meaningful activities can have on the well-being of people living with dementia. This should be considered, for example, when reforming legislation.

## INTRODUCTION

Ever since its outbreak in early 2020, the COVID-19 pandemic has had a huge impact on people’s lives around the world. In many countries, older people have been one of the sections of the population that have been hit the hardest by both the virus and the restrictive measures that have been implemented to fight it. For instance, in Finland, the majority (around 86%) of those who have died of coronavirus have been over 69 years of age as old age is a significant risk factor in terms of the prospects of catching a serious form of coronavirus disease [1]. Furthermore, the pandemic has also had on effect on the daily lives of those older people who have not been infected by the coronavirus. Public health measures such as curfews, stay-at-home recommendations, quarantines, limitations on social activities and hobbies, and prohibitions of visits in care facilities have negatively affected the lives of the older people [2].

As old age may lead to vulnerability due to illness or disability that deteriorates a person’s physical, mental, or social capacity [3, 4], in exceptional circumstances— such as the pandemic— this vulnerability is at the risk of increasing further. This is evident, for example, among people with dementia as previous studies indicate [5–7]. In 2019, Alzheimer Disease International (ADI) estimated that globally there are over 50 million people living with dementia. By 2050, the number is estimated to increase to over 150 million [8]. In Finland, the estimated number of people living with dementia is 193,000. Approximately 14,500 people develop Alzheimer’s or a related disease yearly [9]. However, ADI has estimated that up to 75% of all dementia cases remain undiagnosed worldwide [10]. Furthermore, the pandemic has increased the delay in diagnoses. According to Gauthier et al. [10], 90% of clinicians reported additional delays due to COVID-19.

In Finland, over 90% of those aged 75 and over live in their private homes with help from the family members and/or with home care services [11]. In 2020, there were some 208,000 home care clients in Finland, 71% of whom were 75 years of age and over [12]. Furthermore, the number of people with dementia in informal family care increased nearly 10% between 2012 and 2017 and, in 2017, dementia was already the most common reason for informal family care being needed [13]. The rest, less than 10% of those aged 75 and over, reside in regular or 24-h service housing units, in nursing homes, or in long-term care service in health centers [11]. Roughly half the people residing in 24-h service housing units (53%) and in nursing homes (50%) have some form of dementia [14, 15].

The aim of this study is to find out how the COVID-19 pandemic and related restrictive public health measures have affected the rights and well-being of people living with dementia in Finland. Our particular focus is on the impact of restrictions on mobility and meeting other people. To explore this, we, along with the Alzheimer Society of Finland, conducted a survey of people living with dementia and their family members. The more general aim of the article is to recognize the problems of the current legal regulation on dealing with pandemics. We hope that this study provides valuable information for the legislator on how different restrictions have actually affected the well-being of people with dementia during the pandemic. In October 2020, the Ministry of Justice appointed a working group whose task is to prepare for a full reform of the current Emergency Powers Act. Later, a reform of the Communicable Diseases Act will be initiated. The knowledge about the negative impact of restrictions on people with dementia is vital when reforming the legislation.

We begin with a description of the legal framework and the actual restrictions that were imposed on older people followed by a presentation of the methods and results of our study.

### Legal framework

#### Restrictions on mobility

Section 118 of the Emergency Powers Act (1552/2011) authorizes the Government to issue decrees to temporarily restrict or forbid stays and movements in specified areas or regions if this is deemed necessary for the prevention of a danger seriously threatening the lives or health of the people. Furthermore, Section 23 of the Constitution of Finland (731/1999) authorizes Parliament to pass an act or the Government to issue a decree to impose similar measures. In practice, both provisions allow the imposition of curfews and stay-at-home orders. No mandatory curfews or stay-at-home measures have thus far been imposed in Finland during the pandemic.

However, the Government did restrict domestic travel once during the COVID-19 outbreak, in March 2020. By virtue of section 118 of the Emergency Powers Act, the Government issued a Decree on Temporary Restrictions on Movement to Protect the Population (146/2020) on 27 March 2020. The Decree prohibited traffic and movement between the southernmost province of Uusimaa and the rest of Finland, with some exceptions, such as necessary care of close relatives. The Decree was valid from 28 March to 19 April 2020 and gave rise to complaints to the Parliamentary Ombudsman. Most of these complaints concerned the actions of the police in monitoring the prohibition. For instance, in one particular case a police officer had refused a person to cross the border and to deliver groceries and medicine for their 86-year-old father. The police officer held that the visit was unnecessary because the complainant’s father was able to shop and was regularly visited by a nurse [16].

The restrictions concerning domestic travelling and mobility have mostly been of a recommendatory nature. At the beginning of the pandemic, in March 2020, the Government strongly recommended that all those aged 70 and over should avoid close contact with others and stay at home in quarantine-like conditions [17]. Some municipalities gave short-term recommendations to stay at home and limit contact to a minimum when there were sudden increases in COVID-19 infections locally [18].

#### Restrictions on visits

Section 58 of the Communicable Diseases Act (1227/2016) authorizes the municipal body responsible for the control of communicable diseases to close social and health care units, educational institutions, day care centers, and other similar facilities in its territory. The Regional State Administrative Agencies may make corresponding decisions in their respective jurisdictions when the decisions are needed for an area covering several municipalities. Decisions can only be made for a maximum of one month at a time. This particular provision or any other provision does not, however, give the authorities the right to limit visits to round-the-clock care facilities.

However, it was not at all clear at the beginning of the pandemic whether it was possible to prohibit visits to round-the-clock care facilities. As early as 20 March 2020, the Ministry of Social Affairs and Health issued instructions on COVID-19 related preventive measures in respect of round-the-clock care facilities. These instructions were later updated on 9 April and 15 May 2020 including guidelines on how to prevent infections, for instance, by reference to room arrangements, work shift arrangements, hygiene procedures, and prohibitions on visits [19]. On 16 June 2020, the Ministry issued further instructions on visits to social welfare and health care units during the pandemic [20]. These instructions empowered directors of units to prohibit visits to them if need be. It was claimed that section 17 of the Communicable Diseases Act which requires unit directors, on a general level, to prevent infections in their units provided a legal basis for such prohibitions.

The Ministry’s instructions, as well as individual decisions to prohibit visits, were soon called into question by patients’ family members. Consequently, several administrative complaints were lodged with the Parliamentary Ombudsman. The Deputy Parliamentary Ombudsman concluded that the instructions on visit prohibitions were unlawful and without legal basis.

Unit directors’ decisions to prohibit or restrict visits also led to appeals being lodged with administrative courts. In January 2021, the Supreme Administrative Court gave a ruling in which it came to the same conclusion as the Deputy Parliamentary Ombudsman, i.e., that the Ministry’s instructions were not legally binding, and the decision to restrict meetings between the appellant and the appellant’s father lacked legal basis and was thus unlawful [21].

In conclusion, there is no legal basis for general prohibitions on visits to round-the-clock care facilities in Finland. However, the Finnish Institute for Health and Welfare (*Terveyden ja hyvinvoinnin laitos*, *THL*) has issued guidelines on how to safely carry out visits to such facilities during the pandemic. These guidelines include instructions on hand hygiene, the use of face masks, social distancing and so on.

#### Access to services

Section 51 of the Healthcare Act (1326/2010) provides that the assessment of the need for healthcare services must be made within three working days from the patient’s first contact with the health center. If the need has been established, the treatment must be given within three months. Regarding dental healthcare services, the three-month time limit may be exceeded by an additional three months if this does not endanger the patient’s health. With regard to specialized healthcare services, the service need assessment must be performed within three weeks from the contact, and the necessary treatment provided within six months (Section 52).

Section 36 of the Social Welfare Act (1301/2014) provides that the assessment of a person’s need for social welfare services must be started immediately and finished without unnecessary delay once the person has sought services. If the person is 75 years old or over, the making of the assessment must be started within seven weekdays. Moreover, according to Section 18 of the Act on Supporting the Functional Capacity of the Older Population and on Social and Health Services for Older Persons (980/2012), if the need for a service has been established, the service must be provided without unnecessary delay or within three months at the latest.

Section 88 of the Emergency Powers Act mandates the Government to issue a decree by virtue of which the time limits prescribed in the Healthcare Act and Social Welfare Act can be ignored during a state of emergency. Indeed, a state of emergency was declared in Finland twice during the COVID-19 pandemic— on 16 March 2020 and on 1 March 2021. The first time it lasted three months and the second time two months. During both states of emergency, the Government issued such decrees (127/2020 and 217/2021). This also had a bearing on older people’s access to services. In the 2020 annual report the Parliamentary Ombudsman noted several problems relating to older people’s access to social welfare and healthcare services. For instance, there were problems in organizing adequate services for family carers who take care of older family members and serious delays in the provision of dental healthcare of the older people [22].

## METHODS

To find out how people living with dementia have experienced the public health measures described in the previous section, we carried out a web-based survey (using Surveypal) in spring 2021 in collaboration with the Alzheimer Society of Finland. In addition to people living with dementia, the web-based survey was also aimed at their family members, as persons who live with more advanced dementia (and who were possibly affected most by the restrictions) may not have been able to answer the questions in the survey, and family members often have valuable information about their living conditions to impart. The inclusion criteria were: 1) personal experience of dementia or being close to a person living with dementia, 2) the ability to answer the survey independently or with the assistance of another person. So we asked people with dementia directly about their experiences of the effects of the pandemic. In turn, we asked family members how the pandemic has affected their loved one with dementia or, for example, whether the rights of their loved one with dementia have been restricted.

The Alzheimer Society of Finland, an association that provides help and assistance for people with dementia and their caregivers, implemented the survey. Appropriate ethical procedures of informed consent, avoiding harm and confidentiality were rigorously followed. Formal ethical approval for the study was not sought: According to the research ethics guidelines of Finnish National Board on Research Integrity (TENK), no prior ethical review is required in the case of an anonymous survey for adults, where the response is voluntary and the survey does not, i.e., involve a risk of harm to the respondents or their family members that goes beyond the limits of normal everyday life [23]. The landing page of the survey provided information about the purpose and methods of the study, responding to the survey was completely voluntary, and no direct personal data were collected. Completion of the survey was considered as consent. The survey was open from 7 to 31 May 2021, and was distributed via the Alzheimer Society of Finland’s networks and via various social media channels.

### Survey on well-being and rights during COVID-19 pandemic

Participants completed a total of 13 survey items regarding background variables such as age, gender, and living arrangements (living alone, with a family member, or in a service housing unit). In addition, we asked general questions about well-being during the pandemic, restrictions on freedom, access to services, information on pandemic regulations and guidelines as well as any problems experienced with authorities. The survey included both multiple choice and open-ended questions.

Well-being is, of course, an ambiguous and multifaceted concept. The OECD’s well-being framework recognizes 11 key dimensions of well-being: income and wealth, health, social connections, housing, work and job quality, work life balance, knowledge and skills, civil engagement, safety, subjective well-being, and environment quality [24]. Subjective well-being refers to people’s perceived quality of life. According to a well-established definition, quality of life comprises the following key elements: mental well-being, physical well-being, material well-being, self-determination, personal growth and development, close relationships, social inclusion, and the realization of rights. Together, these aspects affect an individual’s perceived well-being [25]. We asked the respondents to our survey to evaluate the effects of the pandemic on the life and well-being of persons with dementia. First, we asked for an assessment of their current well-being. Next, if respondents felt that the well-being of a person with dementia was moderate, poor, or very poor, we asked a follow-up question about their views on whether it had deteriorated during the pandemic, on a scale of 0 to 10. We then broadened the question on the effects of the pandemic by asking about respondents’ experience of how it may have affected different aspects of their life such as social relationships, mental well-being, physical capacity, and access to social and health care services.

The respondents to our survey were asked multiple questions about restrictions on freedom during the pandemic, i.e., whether their right to meet up with people was restricted, whether their freedom to move outside their homes was restricted, and whether their lives were restricted in other ways. Respondents were also asked in an open-ended question to indicate by whom the restrictions had been imposed.

People living with dementia and their family members were asked whether there was *enough information* available regarding the COVID-19 restrictions, especially in respect of the following issues: what has been permitted or forbidden during the pandemic; which of the restrictions are based on law or, alternatively, on recommendations; and how to run daily errands and to communicate with close family members during the pandemic.

The survey also included questions concerning whether a person living with dementia had encountered any of the following difficulties with social and health care services during the pandemic: difficulties in accessing treatment and care, problems with accessing social services, incorrect entries in patient records, limited access to information, medical malpractice, disrespectful treatment, or otherproblems.

Furthermore, people living with dementia and their family members were asked whether they had identified any *obstacles or problems with the authorities* during the pandemic regarding the following issues: difficulty in contacting the authorities, poor access to information, difficulty in dealing with matters electronically or by phone, difficulty in obtaining information on the progress of one’s own case, difficulty in obtaining advice from the authorities, advice from the authority not being helpful, or other problems. The idea for the questions regarding the problems with social and health care services or dealing with the authorities came from a study focusing on the legal issues experienced by aging populations, which gathered older people’s stories (*n* = 324). Issues concerning healthcare were the most common [26]. Finally, we asked what kind of measures would support people with dementia to recover from the challenges and impacts of the pandemic.

### Participant information and data analysis

We received a total of 199 answers, of which 31 were given by persons with dementia (PwD) and 168 by their family members (FM). The average age of respondents who were people living with dementia was 71.5 (median 70.4). The youngest was 46 years old, and the oldest was 89 years old. As for family members, the average age was 60.3 (median 61), of whom the youngest was 22 years old and the oldest 84 years old. A total of 55% of respondents belonging to the category of people living with dementia (*n* = 17) were male, and 45% were female (*n* = 14). A total of 71% (*n* = 22) of respondents who were people living with dementia answered with assistance, and 29% (*n* = 9) alone. Of respondents that represented family members with dementia, 44% were spouses (*n* = 74), 42% were children (*n* = 70), and 14% were other family members (*n* = 24). About one-fifth (*n* = 41) of the people living with dementia whose situations were reported in this survey (*n* = 199) lived alone in their own home, just under half (*n* = 86) lived at home with a spouse or other family member, about one-quarter (*n* = 49) in a service housing unit, and about one-tenth (*n* = 23) had some other form ofhousing.

As a relatively low number of answers were received from people living with dementia, it is justifiable to examine the answers received both from people with dementia and their family members together. Respondents’ views on well-being and access to rights are described as frequencies. Some answers to open-ended questions are also provided to give a more nuanced picture of their experiences. In this type of internet survey, the sample is not necessarily representative, which is why the results can only be considered indicative.

## RESULTS

### Restrictions on freedom

Restricting a person’s freedom can, of course, mean many things. It may, for example, include preventing a person from entering or leaving a room, defined space, or building, forbidding certain actions, forcing or putting pressure on someone to do something that they do not want to do, or denying them the right to make certain decisions [27].

A conflict between the principles of autonomy and protection usually emerges in cases where a person’s freedom has to be restricted [28]. When restrictions on persons’ freedom are imposed, a balance between these two principles must be struck.

**Fig. 1:**
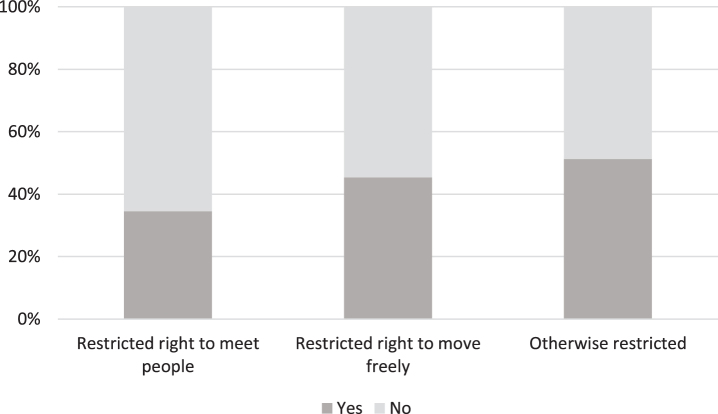
The experiences of people living with dementia and their family members of the restrictions directed at people with dementia during COVID-19 (*n* = 199).

As shown in Fig. 1, over a third (*n* = 68) of respondents (*n* = 199) found that the *right to meet people* was restricted among people living with dementia. Graphs are based on questions with yes or no options.

The responses reveal that some people with dementia had voluntarily restricted meetings in accordance with official recommendations. However, sometimes restrictions were imposed by family members, authorities, social workers, residential care homes and doctors. 43% reported that people with dementia living at home had restricted opportunities to meet other people. For those living in care homes, 86% of respondents reported these restrictions. It was told, for example, that some residential care homes had banned visits altogether. The following responses from family members provide examples of how the ban on visits to care units was implemented:

*(FM79)* ‘*At first, a ban on visits for many months, then in summer 2020 meetings outside were possible, and now during winter in father*’*s room but not in [meeting facilities].*’

*(FM70)* ‘*From spring until death [of a person with dementia], the care unit banned visits inside. The person with dementia in palliative care was only allowed to be visited once, when the person was already unconscious. During summer, it was possible to meet outside, or on the porch during autumn, but not in the meeting facilities inside, or invitations to [their] own room.*’

Almost half (*n* = 88) of the respondents experienced that *freedom of movement* among people with dementia was restricted. Again, while some reported that the movement was restricted by the people with dementia themselves, others said that it was imposed by their family members, authorities, hospital district, and the care home’s management. 44% told that mobility was restricted for people with dementia who live at home. For those living in care homes, 86% of respondents reported these restrictions. The quotes below illustrate how making one’s own decisions about recommendations or ‘negotiating’ how to deal with them has been possible for some people living with dementia but not all:

*(PwD28)* ‘*I have limited my errands, and, among other things, have ordered food online, avoided public transportation, and negotiated ground rules with my wife. Nobody has given direct restriction orders.*’

*(FM46)* ‘*Home visits are not allowed in the care home. If you leave, after returning you will be isolated for two weeks in your own room. Impossible for a person with dementia who is anxious and does not understand the changes. Torture for the person and for their family members.*’

Furthermore, half (*n* = 100) of the respondents noted that *restrictions other than* those on mobility and meeting other people were also imposed on people with dementia. When clarification was requested in an open-ended question, many responses indicated that daytime activities had been terminated at care homes, and physical activities and other hobbies had been paused.

**Fig. 2:**
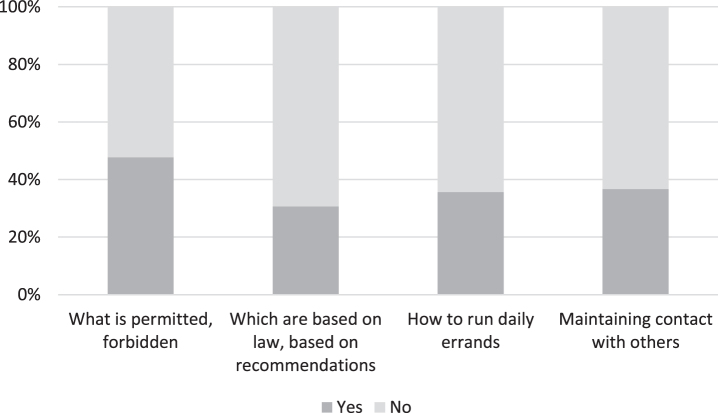
The experiences of people living with dementia and their family members as to the adequacy of information provided about COVID-19 restrictions (*n* = 199).

Roughly half (*n* = 95) of the respondents took the view that there was sufficient information about what was allowed and what was forbidden during the pandemic (see Fig. 2). Over two-thirds of respondents felt that there was insufficient information as to which of the restrictions were based on legal provisions and which were recommended by the authorities. The same shortcomings affected information and instructions on running daily errands and keeping in contact with family members. The following quote exemplifies the difficulties experienced in navigating through complex information and confusing policies:

*(FM6)* ‘*I must admit that during this pandemic, we have relied more on the information provided by the local Alzheimer Association than on the confusing communication between the government, Ministry of Social Affairs and Finnish Institute for Health and Welfare. If only there had been a person who could have filtered and structured the confusing policies of the above-mentioned authorities and shared the information, for example, by e-mail.*’

It is the responsibility of public authorities to provide adequate information on decisions that affect citizens. Our survey is indicative of major shortcomings in terms of information provided on restrictions during the pandemic.

### The pandemic and the well-being of people with dementia

A total of 33% of respondents assessed the current level of well-being of people with dementia as poor or very poor, 47% that it was moderate, and 20% that it was good or very good. Only one respondent indicated that their well-being had not deteriorated during the pandemic. According to 21% (*n* = 30), the well-being of a person with dementia had decreased slightly (1–3), according to 37% (*n* = 53) somewhat (4–6) and, according to 41 % (*n* = 58), it had decreased a lot (7–10).

**Fig. 3:**
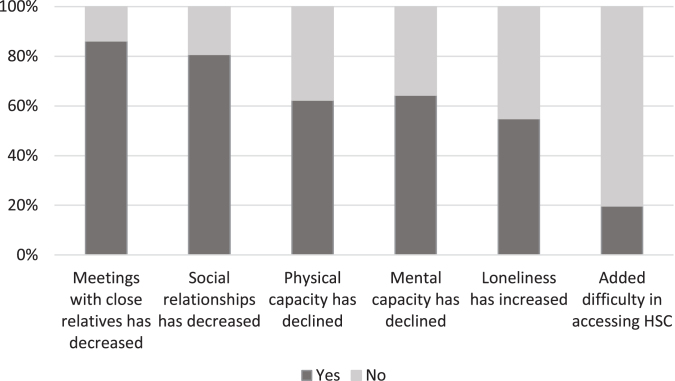
The experiences of people with dementia and their family members as to the impact of COVID-19 on the lives of people with dementia (*n* = 199).

Figure 3 below describes how the pandemic affected the lives of persons with dementia.

Four-fifths (*n* = 171) of the respondents had experienced a decrease in visits between people with dementia and their family members during the pandemic. An equal number (*n* = 160) noted that other social relationships had also declined, and approximately half stated that loneliness among people with dementia had increased. The following quote illustrates how social distancing challenges the maintenance of mental well-being particularly among people with dementia living alone:

*(FM146)* ‘*Yes, the biggest problem for a person living alone, whether with dementia or not, is loneliness. When nothing is organized because of the aim of limiting infections, it is very difficult to keep up good spirits.*’

As mentioned above, half (*n* = 100) of the respondents reported restrictions on daytime activities, physical activities, and hobbies during the pandemic. For example, exercise and outdoor activities are important for people with dementia. Below are some examples provided by our respondents:

*(PwD20)* ‘*Swimming pool, gym, and water gym have been paused. There has been no activity in Muistiluotsi [support center for people with dementia].*’

*(FM33)* ‘*She misses going out but can’t go outside alone. There are not enough people to help with outdoor activities.*’

Two-thirds (64%, *n* = 128) of our respondents reported that the mental well-being of a person living with dementia had declined during the pandemic, as had their physical capacity (62%, *n* = 124). For example, one respondent described how the physical condition of a person with dementia deteriorated during the period of restrictions as follows:

*(FM146)* ‘*My mother’s physical condition collapsed in a year. During the corona year, my mother, who used to walk on her own, has become a frail old woman, stumbling crookedly with the help of a walker. Muscle strength completely gone after a year in her own little room.*’

As Fig. 3 above shows, a fifth of the respondents experienced greater difficulty or delays in accessing social and health services during the pandemic. Respondents’ experiences of access to services are described in more detail in the next section.

The respondents reported that face-to-face activities would be the most helpful (57%, *n* = 114) in recovering from the challenges and impacts of the pandemic, as well as support and activities provided by associations or organizations (47%, *n* = 946). About a third of respondents took the view that the recommencement of municipal or city activities (30%, *n* = 59) would be helpful. Only one-tenth were of the opinion that remote (12%, *n* = 24) or telephone activities (13%, *n* = 25) would be necessary to recover from a pandemic. The following quotes illustrate the necessity of meeting other people and engaging in everyday activities for people with dementia:

*(FM26)* ‘*Face-to-face (without a mask) peaceful meetings several times a week. The lack of nearness is also clear. Holding hands and hugging is really necessary.*’

*(FM47)* ‘*Mother would need meaning in her life, people, and events around her. Even momentary things that would make her feel good.*’

The most common response to the open question on what kind of information, support, or activities would be helpful for people with dementia was, indeed, a return to as normal a life as possible and the recommencement of various activities.

### Access to services among people living with dementia

**Fig. 4:**
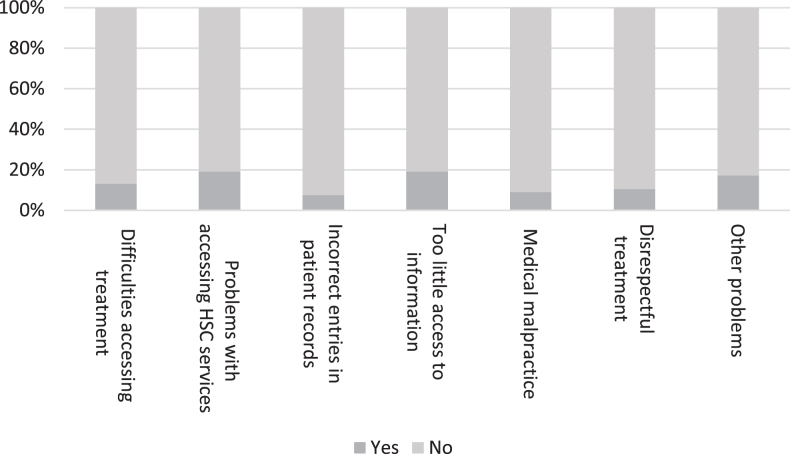
The experiences of people living with dementia and their family members of difficulties in respect of social and health care during COVID-19 (*n* = 199).

As can be observed from Fig. 4, one-fifth (*n* = 38) of the respondents identified problems in accessing social services and adequate information. Only one tenth (*n* = 26) identified issues with accessing treatment and care, incorrect entries in patient records, medical malpractice, unrespectful treatment, or other issues. Half of the respondents (*n* = 100) reported that none of these issues had arisen.

Thus, relatively many had faced some issues with social and health care, in other words, a large proportion reported problems in one of the groups of issues shown in Fig. 4, although only less than fifth reported problems in individual issues. Respondents were given the opportunity to give examples of perceived difficulties in respect of social and health care. Significant problems identified by our respondents related to being left alone to cope with a new life situation after receiving a diagnosis and maintaining one’s well-being during the pandemic. Some examples are provided below:

*(FM80)* ‘*Difficulty getting an appointment for a memory nurse and geriatrician because services have been closed [during the pandemic]*’

*(FM65)* ‘*The biggest challenge is that when the diagnosis came just before the onset of the pandemic, my loved one has not received any personal support, counselling, or counselling for their illness.*’

*(FM171)* ‘*It seems that people living with dementia have been left very much alone with their illness at a time when there was a need for rehabilitative/fitness-maintaining activities.*’

**Fig. 5:**
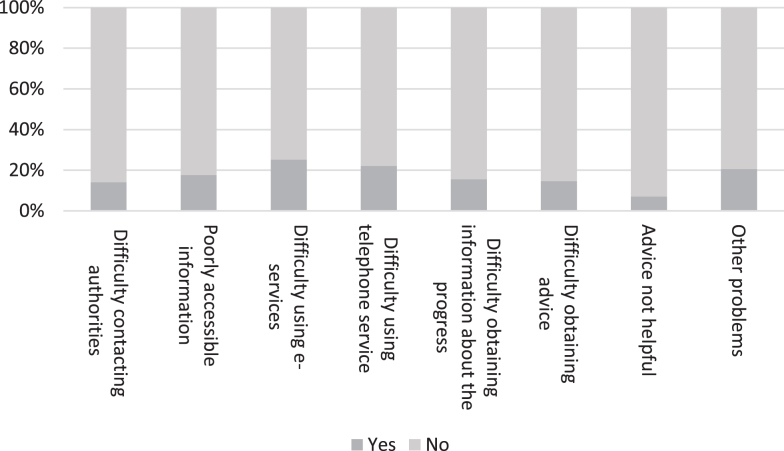
Obstacles and problems faced by a person living with dementia in dealing with authorities during the COVID-19 pandemic (*n* = 199).

As Fig. 5 shows, people with dementia had experienced relatively few difficulties with authorities. Only one-quarter or less of the respondents reported problems in dealing with authorities.

When the respondents were asked if they had experienced any other problems, the most common open response was that a person living with dementia was no longer able to take care of their affairs themselves. As can be seen from the following quotes, loved ones play a key role in such a situation, for example in the context of challenges with digital services or changing care workers. Yet another point raised was the importance of the third sector, in this case Alzheimer associations.

*(FM33)* ‘*It*’*s up to me to fight for her/him. When you aren*’*t always capable of explaining what the problem is, it is important to have the same care worker. Now, the best and the dearest care worker has quit.*’

*(FM198)* ‘*She can’t take care of any of her own affairs, not with a mobile phone, nor with a computer etc. But as a caregiver, I still can take care of them and there have been no difficulties.*’

*(FM39)* ‘*For example, the Alzheimer Society of Finland's Advice Line has been helpful (help and support), but our own municipality has not contacted [us], not even once, during the corona pandemic. The wish would have been to even ask about how we are coping at home.*’

Concerning suitable measures in recovering from the challenges and impacts of the pandemic, one-fifth of respondents took the view that up-to-date information from associations and organizations (21%, *n* = 42), could be helpful. The respondents’ open answers shed further light on the role that third sector organizations play in providing both information and psychosocial support, which seems to be important especially in situations when help is not available elsewhere.

## DISCUSSION

It has been reported across the world that the COVID-19 pandemic has had a major impact on the lives of older people and especially on people living with dementia [29]. This study has explored how the pandemic and related restrictive public health measures have affected the lives of people living with dementia in Finland, with particular focus on the impacts of restrictions on mobility and meeting other people on their well-being and rights.

The results of our study echo the findings of many recent studies, which indicate that the quality of life among people living with dementia has decreased because of the COVID-19 pandemic [5, 6, 7, 30]. According to people with dementia and their family members, by Spring 2021, the pandemic had reduced meaningful activities available to people living with dementia in Finland and decreased the number of meetings and contacts between them and other people. Simultaneously many reported a decline in physical and/or mental well-being, including increases in loneliness (see also, e.g., [31]).

*Meaningful activities* are relevant from the perspective of a person’s well-being [32]. For example, regular exercise and outdoor activities not only help to slow down the changes brought about by aging but also strengthen the mental well-being of the person [7, 33]. Meaningful activity supports a person’s identity [34] and helps maintain the qualities and skills that belong to them and that they enjoy [35]. It can also maintain relationships and social interaction [36]. Recent research suggests that while some people living with dementia seem to be coping well, others experience mental distress, including feelings of loss and isolation, apathy, stress, and anxiety [37, 38]. Some respondents surveyed in our study reported the progression of cognitive impairment that may have resulted from restrictions. It seems that restrictions and the related lack of social engagement challenge the well-being of people living alone the most [39]. Typically, our survey respondents hoped for a return to normal a life as possible and the recommencement of various activities that are shown to contribute to meaningfulness in everyday life among older people [40].

The results of our study are in line with recent studies that have reported the reduction in support from health and social services because of the COVID-19 pandemic [5, 41] such as delays in assessing an older client’s service needs. If the client’s functional capacity deteriorates and the change cannot be monitored, access to adequate help may be delayed. This, in turn, can lead to poor health or nutrition and the need for round-the-clock care [42, 43]. As mentioned in the section describing the legal framework, by virtue of the Emergency Powers Act the government issued decrees according to which lawful maximum waiting periods could be ignored in the context of social welfare and health care services during the two states of emergency. Of course, it is clear that access to care and treatment has worsened even outside the two states of emergency because resources have been temporarily reallocated to the care and treatment of COVID-19 patients. As also mentioned in the legal framework section, in his annual report from 2020 the Parliamentary Ombudsman brought forward several problems related to older people’s access to social welfare and healthcare services in Finland. Our respondents’ open answers included examples of being left alone to cope with a new life situation after receiving a dementia diagnosis or to maintain one’s well-being during the pandemic and the various restrictions.

The results of our study illustrate well the situations a person with dementia faces in their daily life— regardless of the pandemic. If they are unable to take care of their affairs, in many cases their loved ones take care of them; either as an informal or formal caretaker, such as a legal guardian [44]. According to Giebel et al. [45], the task of finding the right services often falls to unpaid carers, which highlights the need for better support both for people with dementia and for their carers to ensure access to services. Supporting well-being among carers is also vitally important in view of the crucial role family and friends have played in maintaining the mental and physical well-being of their loved ones living with dementia during the pandemic [7, 46, 47].

In many countries, there has been a ban on visitors to care homes due to COVID-19. For example, in Canada, care partners have been unable to visit persons with dementia in long-term or palliative care [6]; in Switzerland, the suffering caused by isolation experienced by people living in care homes has been clear to see; and in Germany, many people living with dementia have died alone because family members were not allowed to visit them [29]. Furthermore, people with dementia in care homes in Finland have encountered restrictions concerning their right to meet other people and freedom to leave their place of residence. These restrictions have not been in accordance with the law in all respects. Visiting bans were based on the instructions of the Ministry of Social Affairs and Health which were, however, later found to be unlawful by both the Deputy Parliamentary Ombudsman and the Supreme Administrative Court. As Davies-Abbott et al. [48] note, many people living with dementia in care homes could and should be given the opportunity to participate in decision-making regarding care home adaptations (e.g., how to remain safety and stay connected to family, friends, and the wider world) in response to the pandemic.

The survey interestingly showed how restrictions on the freedom of movement and the right to meet other people were not only imposed by authorities and care unit staff but also by family members/family carers. From the point of view of the people with dementia, this can be problematic because they, especially those with severe dementia, may not have the ability question the restrictions or to recourse to legal redress such as administrative complaints. People with dementia may be exposed to unnecessary restrictions imposed by family members. It is therefore important that in times of crisis such as the COVID-19 pandemic the authorities also give instructions to family carers in which they are reminded of the rights of the those who are cared for to avoid any excessive restrictions.

In this connection, one may also raise the question about the relationship between mandatory restrictions and authorities’ recommendations. Although no mandatory curfew was issued during the pandemic in Finland, a recommendation for 70-year-olds and over to stay at home was given at the beginning of the pandemic, in March 2020. From the point of view of constitutional rights and the principle of proportionality, it is likely that there would not have been legal grounds for a general curfew at that point because it was still early days in the pandemic and there was not enough data on the virus’s fatalness. However, as the virus seemed to affect the older people more severely, it can be considered that such a recommendation was ethically justified. In principle, recommendations work well for people who are able to use their own judgement whether to follow them or not, but in regard to people with dementia and other vulnerable groups, recommendatory measures may just as well expose them to unnecessary restrictions imposed by family carers. This is something that the authorities should also keep in mind when deciding on adequate measures. Separate instructions for family carers on the content and precise meaning of recommendations could be useful.

In general, the results of our study exemplify the *relational* aspects of *autonomy* [49] of people living with dementia. Some of them seem to have more power to influence their own daily lives than others; making one’s own decisions about recommendations or negotiating with one’s family members as to how to deal with them was possible for some people living with dementia but not all. Furthermore, there seems to have been major shortcomings in terms of information on restrictions (see also, e.g., [38]) which left people to navigate through complex information and confusing policies by themselves, although it is the responsibility of public authorities to provide adequate information on decisions that affect citizens. All in all, it is highly important to recognize and acknowledge how interpersonal and institutional interconnections have influenced the freedom of people living with dementia to make decisions and choices regarding their own lives [50] during the pandemic.

The main limitations of our study are its small sample size in general and the fact that collecting data through an online survey may have reduced the number of responses we received from people living with dementia [51]. Thus, the results mainly reflect the experiences of family members. Furthermore, the cross-sectional design of our study does not capture changes over time in the needs and concerns of people with dementia [6]. Thus, more research is needed on the subjective experiences of people living with dementia regarding their rights during the evolving pandemic. Studying the effects of the COVID-19 pandemic from a syndemic perspective [52], for example, would support the identification of interactions between biological, social, and structural factors affecting health and well-being among disadvantaged and marginalized populations during the pandemic [53]. Indeed, this is currently being examined in a Finnish project focusing on pandemic management issues.

The results of this study highlight the importance of bearing in mind the negative effects that restrictions on mobility, meeting other people and meaningful activities can have on the cognitive, physical, and psychosocial well-being of people living with dementia. This should be considered, for example, when reforming legislation. Instead of simply resorting to total restrictions, the legislator should come up with new regulation and mechanisms where, for instance, the right of the person with dementia to meet one’s family and friends could be secured without jeopardizing the person’s or others’ right to health.

## Data Availability

The data supporting the findings of this study may be available on reasonable request from the corresponding author. The data are not publicly available due to privacy or ethical restrictions.
